# The Anti-apoptotic Role of 3′-Untranslational Region in Response to Angiotensin II via Mcl1 Expression

**DOI:** 10.3389/fcell.2020.593955

**Published:** 2021-01-05

**Authors:** Dayin Lyu, Hong Yan, Liyang Chen, Lingmin Zhang, Yanfeng Du, Lexi Ding, Qiulun Lu

**Affiliations:** ^1^Key Laboratory of Cardiovascular and Cerebrovascular Medicine, School of Pharmacy, Nanjing Medical University, Nanjing, China; ^2^Laboratory Medicine Center, The Second Affiliated Hospital of Nanjing Medical University, Nanjing, China; ^3^Eye Center of Xiangya Hospital, Hunan Key Laboratory of Ophthalmology, Central South University, Changsha, China

**Keywords:** anti-apoptosis, Mcl1, Ang II, heart failure, 3′-UTR

## Abstract

Myeloid cell leukemia 1 (Mcl1), an abundant protein in the myocardium, plays an essential role in fibrosis and anti-inflammation in cardiomyocytes to prevent heart failure. However, whether *Mcl1* 3′-untranslated regions (3′-UTR) has the cardio-protecting function remains unclear. Down-regulation of Mcl1 was observed in adult mice heart tissues after Angiotensin II (Ang II) treatment. Consistent with *in vivo* results, the reduction of Mcl1 expression was identified in Ang II-treated neonatal cardiomyocytes. Mechanistically, *Mcl1* 3′-UTR prevented Ang II-induced cardiac apoptosis via up-regulation of Mcl1 and an angiogenic factor with a G-patch domain and a forkhead-associated domain 1 (Aggf1), which plays cardiac-protective role. Our work broadens the scope of gene therapy targets and provides a new insight into gene therapy strategies involving mRNAs’ 3′-UTRs application.

## Introduction

Hypertension contributes significantly to cardiovascular morbidity and mortality (Delles et al., 2010). About 30% of the world population are suffered from hypertension (Choi et al., 2009). As one of the major risk factors for cardiovascular diseases, hypertension causes cardiac dysfunction with cardiac inflammation and fibrosis, accompanied with cardiomyocyte apoptosis (Brooks et al., 2010; Jia et al., 2012). However, the current therapeutic strategies for hypertensive cardiomyopathy are not promising.

Myeloid cell leukemia 1 (Mcl1), a member of an anti-apoptotic Bcl2 family, exhibits functional and structural similarities with anti-apoptotic Bcl2 and Bcl2-xl (Krajewski et al., 1995; Zhou et al., 1997; Rinkenberger et al., 2000; Germain et al., 2011; Thomas and Gustafsson, 2013). Loss of Mcl1 in the adult heart leads to early contractile dysfunction in cardiac hypertrophy, fibrosis and inflammation, resulting in rapid heart failure (Thomas and Gustafsson, 2013; Wang et al., 2013). Although *Mcl1* deficiency did not significantly increase caspase-activation or poly ADP-ribose polymerase (Parp) cleavage, it is critical for normal mitochondrial function (Thomas and Gustafsson, 2013; Wang et al., 2013). Maintaining endogenous levels of Mcl1 in pathological conditions could support clearance of damaged organelles and thus improve cardiac outcomes.

The 3′-UTR as *cis-*regulated element cannot directly regulate mRNA translation and protein expression. However, 3′-UTRs are able to indirectly participate in protein synthesis by bounding to microRNAs (miRNAs) (Treiber et al., 2019). By binding the 3′-UTRs of mRNAs, miRNAs suppress protein synthesis through mRNA degradation or translational repression. In contrast to miRNAs, long non-coding RNAs (lncRNAs), more than 200 nucleotides long, act as competing endogenous RNAs (ceRNAs) to competitively bound with miRNAs to enhance the ability of increasing protein synthesis and mRNA stability of 3′-UTRs (Quinn and Chang, 2016). However, whether *Mcl1* 3′-UTR share the same function in cardio-protection remains unclear.

In this study, we showed the reduction of Mcl1 expression both in hearts from Ang II-infused mice and Ang II-incubated cardiomyocytes. *Mcl1* 3′-UTR strengthening the expression level of Mcl1 indicates that *Mcl1* 3′-UTR, as *trans-*regulatory element, plays a protective role in neo-cardiomyocytes in response to Ang II. We propose that this novel regulatory mechanism could be applied as a novel gene therapy approach for cardiovascular and other diseases.

## Materials and Methods

### Isolation and Culture of Mouse Neonatal Cardiomyocytes

Briefly, hearts harvested from 1–3-days-old mice were digested simultaneously. The collected enzyme solution was centrifuged, followed by discarding of the supernatant. Then, the cardiomyocytes were re-suspended in growth medium with 10% serum. After 24 h, fresh growth medium was added to the cells (Wu et al., 2017). The cells were cultured for 48 h and then incubated with Adeno-associated virus (AAV) either AAV-GFP or AAV-*Mcl1*-UTR (MOI 20) for an additional 12 h.

### Animal Model

Male C57BL/6 mice were housed in the controlled environment with regulation of temperature (22 ± 1°C) and humidity (55%), and unrestricted access to food and water. All animal experiments were in accordance with the principles provided by the National Institute of Health Guideline and were approved by the Animal Care and Use Committee of Central South University (2019sydw0270). Male C57BL/6 mice (21–23 g) were treated with Ang II dissolved in sterile saline at 1 mg/kg/day using subcutaneous osmotic minipumps (Alzet, Cupertino, CA, United States) for 6 weeks (Zhang et al., 2014).

### Adeno-Associated Virus (AAV) Preparation

The AAV9 system was modified and adjusted according to the previous report (Inagaki et al., 2006). DNA sequence of 3′-UTR of mouse *Mcl1* was synthesized from GenScript Corp. Subsequently, the sequence was amplified by PCR. The primers were as follows: *Mcl1*-UTR, forward 5′- atg cgc ggc cgc tct atc tta tta ga -3′ and reverse 5′- atg cgc ggc cgc tgg ggg gaa aaa gg -3′. These PCR products were digested with *Age*I and *Not*I restriction enzyme and subcloned into the AAV9 plasmid. AAV9 was packaged by triple plasmids cotransfection in HEK293 cells and purified as described previously (Pleger et al., 2011).

### Western Blot Analysis

Western blot analysis was carried out with different antibodies as described previously (Qin et al., 2017). Cells were collected in RIPA buffer (Santa Cruz Biotech, TX, United States), and total protein was quantified with BCA assay (Thermo Fisher Scientific, MA, United States). Extracted proteins were then mixed with sample buffer, boiled for 10 min, separated by gel electrophoresis, transferred nitrocellulose membrane (EMD Millipore, Darmstadt, Germany), and blotted with a primary antibody and an appropriate secondary antibody. Primary antibodies and dilutions used were as follows: Mcl1 (1:1000; ab32087, Abcam, Cambridge, United Kingdom) and β-actin (1:1000; Santa Cruz Biotech, TX, United States).

### Quantitative Real-Time PCR Assays

Total RNA from cells was extracted in TRIzol (Life Technologies, CA, United States) according to the manufacturer’s protocol (Li et al., 2019). The cDNA was synthesized by ImProm-II Reverse Transcription System (Promega, WI, United States). The primers of mRNA and Power SYBR Green Master Mix (Life Technologies) were used for real-time PCR assay with lightCycler1.5 Instrument (Roche, Mannheim, Germany). Experiments were performed in triplicate.

### TUNEL Assay

Mouse neonatal cardiomyocytes were treated, used for a TUNEL assay using the *in situ* Cell Death Detection Kit (Roche Diagnostics GmbH). The images were visualized and captured under a fluorescence microscope. More than eight fields in four different sections were examined for each mouse by a researcher who was blinded to the treatments. The percentage of TUNEL-positive cells of the total number of nuclei as determined by DAPI staining (blue) was analyzed. Heart sections were incubated with the labeling solution, but without terminal transferase were used as negative controls (Yao et al., 2017).

### Immunofluorescence

Hearts were excised from mice, embedded in OCT, sectioned into slices, and then fixed with acetone for 10 min. The slides were blocked with goat serum, and incubated with primary antibody (anti-Mcl1, ab32087 from Abcam) overnight. After PBS washed, these slides were incubated with secondary antibody. DAPI was used to counterstain the nuclei (Yao et al., 2017).

### Statistics

Statistical analyses were performed using Prism 8 (GraphPad) software. All data were expressed as means ± SD. Two-group comparisons were analyzed by a Student’s *t* test or non-parametric Wilcoxon rank test whenever appropriate. For comparisons of more than two groups, one-way ANOVA or the generalized linear regression approach was employed for normal distributions and the Kruskal Wallis test for non-normal or small samples. *p* < 0.05 was considered significant.

## Results

### Reduction of Mcl1 Expression in Ang II-Infused Hearts

**FIGURE 1:**
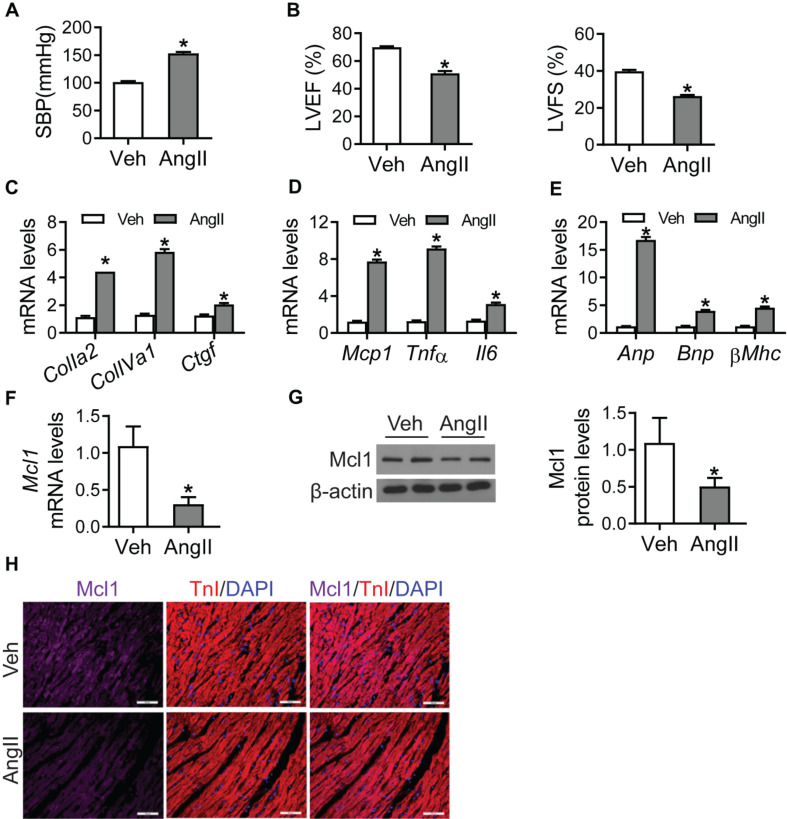
Reduction of Mcl1 expression in Ang II-infused hearts. 8 weeks old mice were infused with vehicle or Ang II (1 mg/kg/day, Sigma) for 6 weeks (*n* = 10 each group). **(A)** Systolic blood pressure (SBP) was measured by the non-invasive tail-cuff method using plethysmography (*n* = 10, * vs. Veh, *p* < 0.05). **(B)** The values of LVEF and LVFS (*n* = 10, * vs. Veh, *p* < 0.05). **(C)** Realtime-PCR analysis for markers for cardiac fibrosis, including ColIa2, ColIVa1, and Ctgf (*n* = 6, * vs. Veh, *p* < 0.05). **(D)** Realtime-PCR analysis for markers for cardiac inflammation, including *Mcp1*, *Tnf*α, and *Il6* (*n* = 6, * vs. Veh, *p* < 0.05). **(E)** Realtime-PCR analysis for markers for heart failure, including *Anp*, *Bnp*, and β*Mhc* (*n* = 6, * vs. Veh, *p* < 0.05). **(F)** Real-time PCR analysis for *Mcl1* mRNA levels (*n* = 6, * vs. Veh, *p* < 0.05). **(G)** Western blot analysis for Mcl1 expression after Ang II infusion (*n* = 6, * vs. Veh, *p* < 0.05). **(H)** Immunofluorescent staining for Mcl1 with heart tissues from mice with Veh/Ang II infused mice (*n* = 6, * vs. Veh, *p* < 0.05).

In order to demonstrate the role of Mcl1 in heart failure, we generated hypertrophic mice infused with Ang II for 6 weeks. Consistent with previously reported, the systolic blood pressure (SBP) of Ang II-infused group was significantly increased (Figure 1A). Echocardiography showed that both left ventricular ejection fraction (LVEF) and left ventricular fractional shortening (LVFS) in Ang II-infused mice were significantly decreased compared with vehicle group (Figure 1B). Meanwhile, the mRNA levels of the markers for cardiac fibrosis, such as *ColIa2*, *ColIVa1*, and *Ctgf*, were increased in hearts from Ang II-infused mice (Figure 1C). And inflammation-related genes *Mcp1*, *Tnf*α, and *Il6* and heart failure genes *Anp*, *Bnp*, and β*Mhc* were up-regulated in the condition of Ang II infusion versus vehicle group (Figure 1D,E). These data showed that Ang II caused cardiac fibrosis and inflammation response leading to heart failure.

Realtime-PCR assay indicated that Ang II caused the reduction of *Mcl1* mRNA levels in heart tissues, compared with vehicle treatment (Figure 1F). Additionally, the decline for Mcl1 protein levels was observed in the heart tissues from Ang II-infused mice compared with vehicle-treated mice (Figure 1G). And immunofluorescent analysis showed that the protein levels of Mcl1 were decreased in heart tissues after Ang II infusion (Figure 1H). Overall, these results suggested that Ang II caused the reduction of Mcl1 in heart tissues.

### Decrease for Mcl1 Expression in Cardiomyocytes After Ang II Incubation

**FIGURE 2:**
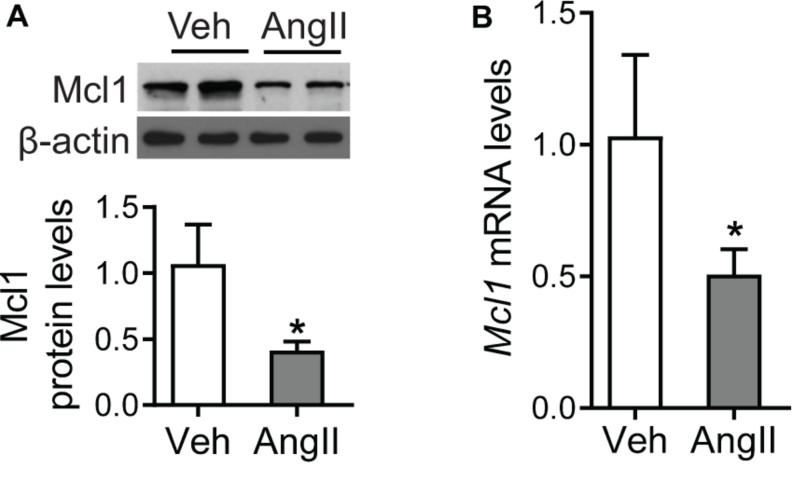
Decrease for Mcl1 expression in cardiomyocytes after Ang II incubation. The purified mouse neo-cardiomyocytes were incubated with vehicle or Ang II (1 μmol). **(A)** Western blot analysis for Mcl1 expression after incubated with vehicle or Ang II (*n* = 6, * vs. Veh, *p* < 0.05). **(B)** Real-time PCR analysis for *Mcl1* mRNA levels (*n* = 6, * vs. Veh, *p* < 0.05).

Although the reduction of Mcl1 was identified in Ang II-infused hearts *in vivo*, the expression level of Mcl1 in response to Ang II was not clear in cardiomyocytes. Since the reduction of Mcl1 expression was associated with severity of cardiac dysfunction, we detected the Mcl1 protein levels using Western blot analysis in Ang II-incubated neonatal cardiomyocytes. The results showed that Ang II decreased Mcl1 protein levels in cardiomyocytes (Figure 2A). Consistently, the results from real-time PCR assay in Figure 2B indicated that *Mcl1* mRNA levels were declined in cardiomyocytes after co-culture with Ang II. These data suggested that *in vitro* Ang II decreased Mcl1 expression levels in cardiomyocytes.

### Up-Regulation of Mcl1 by Ectopic Expression of *Mcl1* 3′-UTR

**FIGURE 3:**
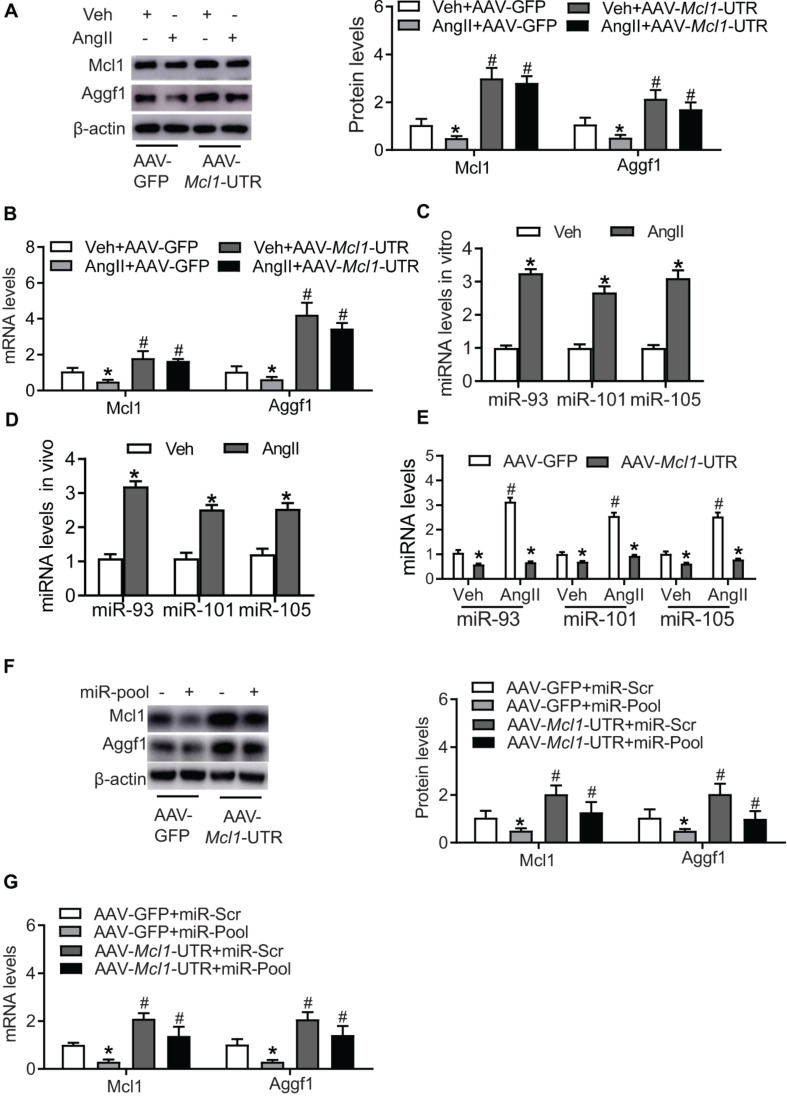
Up-regulation of Mcl1 by ectopic expression of *Mcl1* 3′-UTR. The purified mouse neo-cardiomyocytes were pre-cultured with AAV-GFP or AAV-*Mcl1*-UTR, and then incubated with vehicle or Ang II. **(A)** Western blot analysis for Mcl1and Aggf1 expression after treatment (*n* = 3, * vs. Veh, *p* < 0.05; # vs. AAV-GFP treatment, *p* < 0.05). **(B)** Real-time PCR analysis for *Mcl1* and *Aggf1* mRNA levels (*n* = 3, * vs. Veh, *p* < 0.05; # vs. AAV-GFP treatment, *p* < 0.05). **(C)** The levels of miRNA were analyzed in hearts from Ang II-infused mouse. The heart tissues were collected from mice infused with vehicle or Ang II for 6 weeks (*n* = 3, * vs. Veh, *p* < 0.05). **(D)** The expression levels of miRNA were analyzed in Ang II-treated neo-cardiomyocytes (*n* = 3, * vs. Veh, *p* < 0.05). **(E)** The expression levels of miRNA were detected in cardiomyocytes cultured with AAV-*Mcl1*-UTR (*n* = 3, * vs. Veh, *p* < 0.05; # vs. AAV-GFP treatment, *p* < 0.05). **(F,G)** The levels of Aggf1 and Mcl1 expression were measured after miR-pool expression (miR-93/101/105) in AAV-*Mcl1*-UTR-cultured cardiomyocytes (*n* = 3, * vs. miR-scr, *p* < 0.05; # vs. AAV-GFP treatment, *p* < 0.05).

Due to the cardiac-protective role of Mcl1, to increase Mcl1 level is a prominent candidate for cardiac disease therapy. In order to avoid the side-effective of coding sequences delivery, the non-coding sequence on 3′-UTR of *Mcl1* mRNA was inserted into AAV-delivery system (AAV-*Mcl1*-UTR), ectopically expressing *Mcl1* 3′-UTR. Western blot and real-time PCR analysis showed that the ectopic expression of *Mcl1* 3′-UTR results in increased Mcl1 expression at both protein and mRNA levels (Figures 3A,B).

Previous study reported that *Aggf1* 3′-UTR causes the increase of Mcl1 expression, we explored *Mcl1* 3′-UTR potential regulation on Aggf1 expression. The expression of Aggf1 was detected after *Mcl1* 3′-UTR expression, which suggests the ectopic expression of *Mcl1* 3′-UTR increased the mRNA and protein levels of Aggf1 (Figures 3A,B). Herein, we found that *Mcl1* 3′-UTR increased both of Mcl1 and Aggf1 expression.

### *Mcl1* 3′-UTR Regulates Mcl1 and Aggf1 Expression via MicroRNAs

As the miRNA profile is significantly changed in response to Ang II treatment, the expression levels of numerous miRNAs are up-regulated or down-regulated. Additionally, certain miRNAs recently reported recognize and are bound to 3′-UTRs both of *Mcl1* and *Aggf1* mRNAs, and then affect Mcl1 and Aggf1 expression, such as miR-93, miR-101, and miR-105. The expression of these miRNAs in hearts from Ang II-infused mouse was determined, showing Ang II infusion caused the increased levels of miR-93, miR-101, and miR-105 in hearts (Figure 3C). Consistently, the elevated levels of these miRNAs were observed in cardiomyocytes cultured with Ang II (Figure 3D). The reduction of miR-93, miR-101, and miR-105 was observed after *Mcl1* 3′-UTR overexpression (Figure 3E). Overexpression of miR-pool with miR-93, miR-101, and miR-105 suppressed the increased levels of Mcl1 and Aggf1 both at protein and mRNA levels, indicating *Mcl1* 3′-UTR affects Mcl1 and Aggf1 expression via miR-93/101/105 reductions (Figures 3F,G). These data suggested that *Mcl1* 3′-UTR indirectly regulated Mcl1 and Aggf1 expression.

### The Anti-apoptosis of *Mcl1* 3′-UTR in Ang II-Treated Cardiomyocytes

**FIGURE 4:**
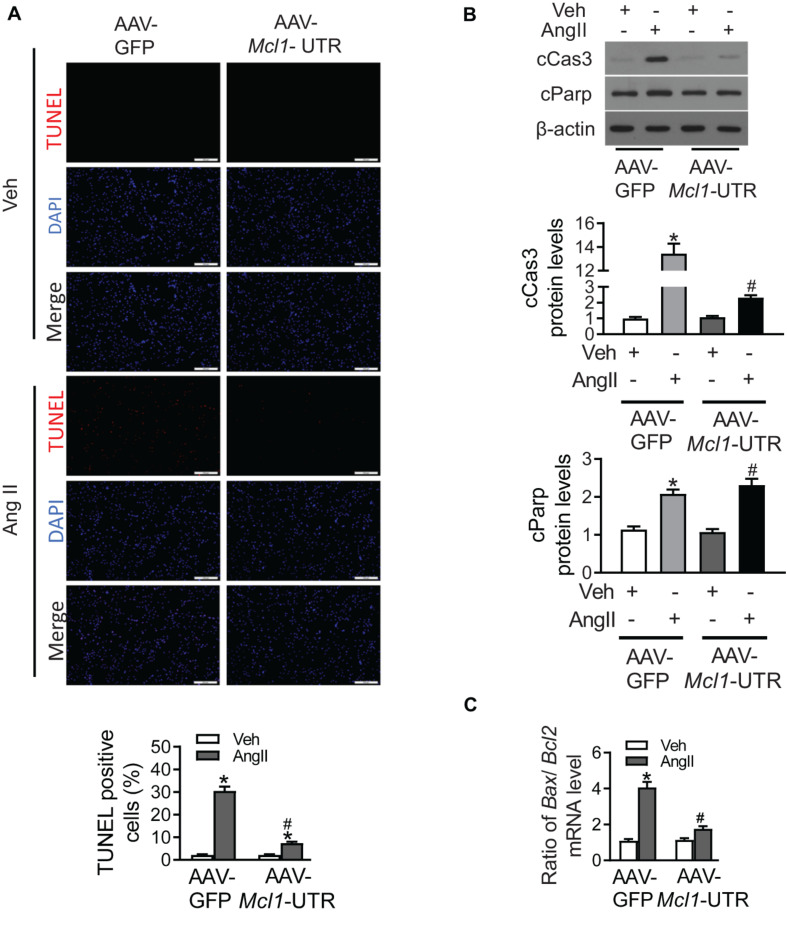
The anti-apoptosis of *Mcl1* 3′-UTR in Ang II-treated cardiomyocytes. The purified mouse neo-cardiomyocytes were co-cultured with AAV-GFP or AAV-*Mcl1*-UTR, and then incubated with vehicle or Ang II. **(A)** The representative images of TUNEL staining were shown (*n* = 6, * vs. Veh, *p* < 0.05; # vs. AAV-GFP treatment, *p* < 0.05). **(B)** Western blot analysis for apoptotic markers, such as cleaved Parp, and cleaved Caspase 3. **(C)** Ratio for *Bax* to *Bcl2* mRNA levels (*n* = 6, * vs. Veh, *p* < 0.05; # vs. AAV-GFP treatment, *p* < 0.05).

Due to up-regulation of Mcl1 levels after the ectopic expression of *Mcl1* 3′-UTR, cardiac apoptosis was detected in Ang II-treated neonatal cardiomyocytes. AAV-*Mcl1*-UTR treatment successfully decreased the upregulated number of TUNEL-positive cells in response to Ang II incubation compared to AAV-GFP treatment (Figure 4A). Consistent with TUNEL results, effects of AAV-*Mcl1*-UTR on Ang II-induced apoptosis in mouse neo-cardiomyocytes were also manifested in the reductions in cleaved PARP (cPARP) and cleaved caspase-3 (cCAS3) (Figure 4B). The ratios of *Bax* mRNA to *Bcl2* mRNA were significantly suppressed by AAV-*Mcl1*-UTR compared with AAV-GFP in neo-cardiomyocytes incubated with Ang II (Figure 4C). Therefore, these data determined that AAV-*Mcl1*-UTR could increase the expression of Mcl1 both in protein level or mRNA level. And *Mcl1* 3′-UTR displays protective role in respond to Ang II-incubated cardiomyocytes by inhibiting apoptosis of cardiomyocytes.

## Discussion

In this study, we found that the reduction of Aggf1 along with alleviated Mcl1 in hearts from Ang II-infused mice and isolated cardiomyocytes treated with Ang II. In neo-cardiomyocytes, Ang II-induced apoptosis was alleviated by the ectopic expression of *Mcl1* 3′-UTR, accompanied with the increased levels of Mcl1 and Aggf1 expression. This indicated that non-coding 3′-UTR as *cis*-regulatory element played an anti-apoptotic role in cardiomyocytes. Interestingly, non-coding sequences could be considered as gene therapeutic targets for cardiovascular diseases, and it would be a novel candidate for other diseases treatments (Yuan et al., 2019).

Both *in vivo* and *in vitro* experiments show that Ang II causes the decline for Mcl1 expression in cardiomyocytes. Ang II causes decreased Mcl1 expression both at protein and mRNA levels in heart tissues as well as isolated myocytes. As previously reported, Mcl1 expression is decreased by miR-204 overexpression *via* binding of the *Mcl1* 3′-UTR, subsequently resulting in the decrease pancreatic cell viability (Chen et al., 2013). MiR-29b over-expression reduces Mcl1 protein levels and increases the resistance to tumor necrosis factor-related apoptosis-inducing ligand (Trail)-caused cytotoxicity (Chen et al., 2013). AR-A014418-induced Mcl1 down-regulation remarkably elicits apoptosis of U937 cells. Furthermore, AR-A014418-elicited ERK inactivation causes the reduction of Mcl1 expression due to inhibition of Mnk1-mediated eIF4E phosphorylation (Lee et al., 2020). In response to stimulus, the levels of Mcl1 expression are decreased, suggesting that the reduction of Mcl1 is associated with the severity of cellular damage and dysfunction.

Non-coding *Mcl1* 3′-UTR can alleviate Ang II-caused cardiac damage. Our results showed that the ectopic expression of a non-coding sequence with *Mcl1* 3′-UTR prevented Ang II-induced apoptosis in neonatal cardiomyocytes. The mechanism underlying *Mcl1* 3′-UTR protecting Ang II-treated cardiomyocytes is through up-regulation of Mcl1 expression. Conversely, cardiac-specific ablation of *Mcl1* results in a rapidly fatal, dilated cardiomyopathy manifested by loss of abnormal myocyte structure (Wang et al., 2013). Meanwhile, in inducible cardiac-specific *Mcl1* knockout mice, *Mcl1* loss rapidly causes cardiomyopathy and death (Thomas et al., 2013). *Mcl1* deficiency may primarily trigger the induction of cardiomyocyte apoptosis, leading to loss of myofibers and the rapid cardiac dysfunction that results in the death of the mice. However, it is possible that the mitochondrial dysfunction induced by Mcl1 loss results in mitochondrial stress that drives autophagy to alleviate the damage. Due to the cardio-protective role of Mcl1, delivery of *Mcl1* 3′-UTR is an effective therapeutic strategy for heart diseases.

The mechanism underlying how *Mcl1* 3′-UTR regulates Mcl1 expression is due to microRNAs. We previously reported that the cluster microRNAs, including miR-93, miR-101, and miR-105, can recognize and bind with the 3′-UTR of *Mcl1* and *Aggf1* mRNAs (Ding et al., 2020). *In vitro* characterization corroborated Aggf1 as a Mcl1 ceRNA and showed that these genes share 3′-UTR binding sites for the same miRNAs, including miR-105, miR-101, and miR-93, which were upregulated in response to Ang II. The ectopic expression of *Mcl1* 3′-UTR caused the reduction of miR-105, miR-101, and miR-93. Due to the declined levels of these miRNA, the mRNA levels of *Aggf1* and *Mcl1* were increased. These results indicated the cardio-protective of *Mcl1* 3′-UTR is independent of Mcl1 regulation.

The advantages of non-coding sequences provide prospective opportunities to be applied in clinics. In the past, non-coding sequences were considered as evolutionary junk, but accumulated evidences suggested that non-coding sequences take part in functional regulatory molecules to mediate cellular processes including chromatin remodeling, transcription, post-transcriptional modifications and signal transduction (Beermann et al., 2016). Non-coding sequences, including microRNAs (miRNAs), long non-coding RNAs (lncRNAs), and circular RNAs (cirRNAs), possess a series of merits than other molecules. Firstly, transcripts of non-coding sequences can be packaged into viral vectors and delivered into targeted cells to mediate their therapeutic effect. Additionally, non-coding sequences have also been gaining more attention given their advantages for dosage control and low immunogenicity. Last but not least, non-coding sequences indirectly take part in gene transcription and keep stable structures better than the therapeutic approaches of proteins and mRNAs.

Our study demonstrates that *Mcl1* 3′-UTR, a non-coding sequence, increases the Mcl1 expression both at mRNA and protein levels in response to Ang II and plays an essential protective role in cardiomyocytes by alleviating the apoptosis of cardiomyocytes. Thus, our data suggest that the ectopic expression of mRNA 3′-UTRs is a novel gene therapeutic approach for cardiovascular and other diseases.

## Data Availability Statement

The raw data supporting the conclusions of this article will be made available by the authors, without undue reservation.

## Ethics Statement

The animal study was reviewed and approved by the Animal Care and Use Committee of Central South University.

## Author Contributions

QL and LD designed the study. DL, HY, LC, LZ, and YD conducted searches, extracted, and analyzed the data. DL and LD wrote the manuscript. All authors contributed to the article and approved the submitted version.

## Conflict of Interest

The authors declare that the research was conducted in the absence of any commercial or financial relationships that could be construed as a potential conflict of interest.

## References

[B1] Beermann J. Piccoli M. T. Viereck J. Thum T. (2016). Non-coding RNAs in development and disease: background, mechanisms, and therapeutic approaches. *Physiol. Rev.* 96 1297–1325. 10.1152/physrev.00041.2015 27535639

[B2] Brooks W. W. Shen S. S. Conrad C. H. Goldstein R. H. Bing O. H. (2010). Transition from compensated hypertrophy to systolic heart failure in the spontaneously hypertensive rat: structure, function, and transcript analysis. *Genomics* 95 84–92. 10.1016/j.ygeno.2009.12.002 20006699

[B3] Chen Z. Sangwan V. Banerjee S. Mackenzie T. Dudeja V. Li X. (2013). miR-204 mediated loss of Myeloid cell leukemia-1 results in pancreatic cancer cell death. *Mol. Cancer* 12:105. 10.1186/1476-4598-12-105 24025188 PMC3848798

[B4] Choi S. M. Seo M. J. Kang K. K. Kim J. H. Ahn B. O. Yoo M. (2009). Beneficial effects of the combination of amlodipine and losartan for lowering blood pressure in spontaneously hypertensive rats. *Arch. Pharm. Res.* 32 353–358. 10.1007/s12272-009-1307-x 19387578

[B5] Delles C. McBride M. W. Graham D. Padmanabhan S. Dominiczak A. F. (2010). Genetics of hypertension: from experimental animals to humans. *Biochim. Biophys. Acta* 1802 1299–1308. 20035862 10.1016/j.bbadis.2009.12.006 PMC2977068

[B6] Ding L. Lu S. Zhou Y. Lyu D. Ouyang C. Ma Z. (2020). The 3′ untranslated region protects the heart from angiotensin II-induced cardiac dysfunction via AGGF1 expression. *Mol. Ther.* 28 1119–1132. 10.1016/j.ymthe.2020.02.002 32061268 PMC7132794

[B7] Germain M. Nguyen A. P. Le Grand J. N. Arbour N. Vanderluit J. L. Park D. S. (2011). MCL-1 is a stress sensor that regulates autophagy in a developmentally regulated manner. *EMBO J.* 30 395–407. 10.1038/emboj.2010.327 21139567 PMC3025469

[B8] Inagaki K. Fuess S. Storm T. A. Gibson G. A. McTiernan C. F. Kay M. A. (2006). Robust systemic transduction with AAV9 vectors in mice: efficient global cardiac gene transfer superior to that of AAV8. *Mol. Ther.* 14 45–53. 10.1016/j.ymthe.2006.03.014 16713360 PMC1564441

[B9] Jia L. Li Y. Xiao C. Du J. (2012). Angiotensin II induces inflammation leading to cardiac remodeling. *Front. Biosci.* 17 221–231. 10.2741/3923 22201740

[B10] Krajewski S. Bodrug S. Krajewska M. Shabaik A. Gascoyne R. Berean K. (1995). Immunohistochemical analysis of Mcl-1 protein in human tissues. Differential regulation of Mcl-1 and Bcl-2 protein production suggests a unique role for Mcl-1 in control of programmed cell death in vivo. *Am. J. Pathol.* 146 1309–1319. 7778670 PMC1870904

[B11] Lee Y. C. Shi Y. J. Wang L. J. Chiou J. T. Huang C. H. Chang L. S. (2020). GSK3beta suppression inhibits MCL1 protein synthesis in human acute myeloid leukemia cells. *J. Cell Physiol.* 236 570–586. 10.1002/jcp.29884 32572959

[B12] Li J. Gong L. Liu S. Zhang Y. Zhang C. Tian M. (2019). Adipose HuR protects against diet-induced obesity and insulin resistance. *Nat. Commun.* 10:2375. 10.1038/s41467-019-10348-0 PMC6542850 31147543

[B13] Pleger S. T. Shan C. Ksienzyk J. Bekeredjian R. Boekstegers P. Hinkel R. (2011). Cardiac AAV9-S100A1 gene therapy rescues post-ischemic heart failure in a preclinical large animal model. *Sci. Transl. Med.* 3:92ra64. 10.1126/scitranslmed.3002097 21775667 PMC4095769

[B14] Qin X. Liu S. Lu Q. Zhang M. Jiang X. Hu S. (2017). Heterotrimeric G stimulatory protein alpha subunit is required for intestinal smooth muscle contraction in mice. *Gastroenterology* 152 1114.e5–1125.e5. 28043906 10.1053/j.gastro.2016.12.017 PMC7430528

[B15] Quinn J. J. Chang H. Y. (2016). Unique features of long non-coding RNA biogenesis and function. *Nat. Rev. Genet.* 17 47–62. 10.1038/nrg.2015.10 26666209

[B16] Rinkenberger J. L. Horning S. Klocke B. Roth K. Korsmeyer S. J. (2000). Mcl-1 deficiency results in peri-implantation embryonic lethality. *Genes Dev.* 14 23–27. 10640272 PMC316347

[B17] Thomas R. L. Gustafsson A. B. (2013). MCL1 is critical for mitochondrial function and autophagy in the heart. *Autophagy* 9 1902–1903. 10.4161/auto.26168 24165322 PMC4028340

[B18] Thomas R. L. Roberts D. J. Kubli D. A. Lee Y. Quinsay M. N. Owens J. B. (2013). Loss of MCL-1 leads to impaired autophagy and rapid development of heart failure. *Genes Dev.* 27 1365–1377. 10.1101/gad.215871.113 23788623 PMC3701192

[B19] Treiber T. Treiber N. Meister G. (2019). Publisher correction: regulation of microRNA biogenesis and its crosstalk with other cellular pathways. *Nat. Rev. Mol. Cell Biol.* 20:321. 10.1038/s41580-019-0106-6 30728477

[B20] Wang X. Bathina M. Lynch J. Koss B. Calabrese C. Frase S. (2013). Deletion of MCL-1 causes lethal cardiac failure and mitochondrial dysfunction. *Genes Dev.* 27 1351–1364. 10.1101/gad.215855.113 23788622 PMC3701191

[B21] Wu S. Lu Q. Wang Q. Ding Y. Ma Z. Mao X. (2017). Binding of FUN14 domain containing 1 with inositol 1,4,5-trisphosphate receptor in mitochondria-associated endoplasmic reticulum membranes maintains mitochondrial dynamics and function in hearts in vivo. *Circulation* 136 2248–2266. 10.1161/circulationaha.117.030235 28942427 PMC5716911

[B22] Yao Y. Lu Q. Hu Z. Yu Y. Chen Q. Wang Q. K. (2017). A non-canonical pathway regulates ER stress signaling and blocks ER stress-induced apoptosis and heart failure. *Nat. Commun.* 8:133. 10.1038/s41467-017-00171-w PMC5527107 28743963

[B23] Yuan Y. Wang J. Chen Q. Wu Q. Deng W. Zhou H. (2019). Long non-coding RNA cytoskeleton regulator RNA (CYTOR) modulates pathological cardiac hypertrophy through miR-155-mediated IKKi signaling. *Biochim. Biophys. Acta Mol. Basis Dis.* 1865 1421–1427. 10.1016/j.bbadis.2019.02.014 30794866

[B24] Zhang W. Wang Q. Wu Y. Moriasi C. Liu Z. Dai X. (2014). Endothelial cell-specific liver kinase B1 deletion causes endothelial dysfunction and hypertension in mice in vivo. *Circulation* 129 1428–1439. 10.1161/circulationaha.113.004146 24637557 PMC3972325

[B25] Zhou P. Qian L. Kozopas K. M. Craig R. W. (1997). Mcl-1, a Bcl-2 family member, delays the death of hematopoietic cells under a variety of apoptosis-inducing conditions. *Blood* 89 630–643. 10.1182/blood.v89.2.630 9002967

